# miR-27a and miR-27a* contribute to metastatic properties of osteosarcoma cells

**DOI:** 10.18632/oncotarget.3025

**Published:** 2015-02-28

**Authors:** Zaidoun Salah, Rand Arafeh, Vadim Maximov, Marco Galasso, Saleh Khawaled, Samah Abou-Sharieha, Stefano Volinia, Kevin B. Jones, Carlo M. Croce, Rami I. Aqeilan

**Affiliations:** ^1^ The Lautenberg Center for Immunology and Cancer Research, IMRIC, Hebrew University-Hadassah Medical School, Jerusalem, Israel; ^2^ Al Quds-Bard College, Al-Quds University, Abu Dies, East Jerusalem, Palestine; ^3^ Biosystems Analysis, LTTA, Department of Morphology, Surgery and Experimental Medicine, Università degli Studi, Ferrara, Italy; ^4^ Department of Molecular Virology, Immunology and Medical Genetics, The Ohio State University Wexner Medical Center, Columbus, USA; ^5^ Huntsman Cancer Institute at the University of Utah, Salt Lake City, USA

**Keywords:** osteosarcoma, metastasis, miR-27a, CBFA2T3, star miRNA

## Abstract

Osteosarcoma (OS) is the most common primary malignant bone tumor in adolescents and young adults. The essential mechanisms underlying osteosarcomagenesis and progression continue to be obscure. MicroRNAs (miRNAs) have far-reaching effects on the cellular biology of development and cancer. We recently reported that unique miRNA signatures associate with the pathogenesis and progression of OS. Of particular interest, we found that higher expression of *miR-27a* is associated with clinical metastatic disease. We report here that overexpression of miR-27a/miR-27a*, a microRNA pair derived from a single precursor, promotes pulmonary OS metastases formation. By contrast, sequestering miR-27a/miR-27a* by sponge technology suppressed OS cells invasion and metastases formation. miR-27a/miR-27a* directly repressed *CBFA2T3* expression among other target genes. We demonstrated that *CBFA2T3* is downregulated in majority of OS samples and its over expression significantly attenuated OS metastatic process mediated by miR-27a/miR-27a* underscoring *CBFA2T3* functions as a tumor suppressor in OS. These findings establish that miR-27a/miR-27a* pair plays a significant role in OS metastasis and proposes it as a potential diagnostic and therapeutic target in managing OS metastases.

## INTRODUCTION

Bone and joints cancer is the third cause of cancer death in childhood and adolescence. Osteosarcoma (OS) is the major type of bone and joint cancer [1]. Significant improvement in overall survival of OS patients was achieved after implementation of relatively effective chemotherapy in 1970s, but a third of patients still die during 5 years after diagnosis [2]. Overall 5 years survival drops even further to about 30% for OS patients with metastases at diagnosis [3]. Therefore, improvements in therapy of OS patients, particularly, those with the metastatic disease are needed. Advances in OS treatment are inconceivable without better understanding of molecular mechanisms of osteosarcomagenesis and, especially, metastatic processes in this kind of cancer.

MiRNAs are small RNA molecules ranging in size from 16 to 27 bases; usually between 21 and 23 bases for most miRNAs [4]. Although diverse mechanisms of gene expression regulation by miRNAs have been demonstrated [5], they mostly repress gene expression at the posttransciptional level [6]. Mature miRNAs arise from a multistep process. Primary miRNAs (pri-miRNAs) are transcribed by polymerase II in most cases. Pri-miRNAs are processed by the nuclear RNase III Drosha yielding hairpin-like precursors of miRNAs (pre-miRNAs), which are then transported from the nucleus to the cytoplasm. Pre-miRNAs are then sliced by the cytoplasmic RNase III Dicer resulting in small double strand RNAs (dsRNAs). Guide strands are immediately bound by Argonaute proteins and recruited to the RNA-induced silencing complex (RISC), where these guide strands function as mature miRNAs. The other, so-called “passenger” or “star”, strand is considered to be mostly degraded in the majority of cases [7]. However, mounting evidence from genome-wide studies indicate that star miRNAs are not only widely present in different types of mammalian cells but also functional [8, 9].

MiRNAs are commonly deregulated in cancer and involved in all aspects of carcinogenesis [10–12]. Of particular interest, many miRNAs regulate the metastatic process either promoting or suppressing it [13, 14]. Notably, evidences are being accumulated that deregulation of star miRNAs expression also plays causative role in cancer including the metastatic process [15–18].

Previously, we reported an osteosarcoma-specific microRNA signature. Of particular interest, we found higher expression of *miR-27a* and miR-181c* that is associated with clinical metastatic disease [19]. Here, we demonstrate that the miR-27a/miR-27a* pair play pro-metastatic roles in OS cells that is mediated, at least in part, by targeting *CBFA2T3*, a putative tumor suppressor. Indeed, we provide evidence that the *CBFA2T3* gene behaves as a tumor suppressor in OS. In addition, we show that expression of other miR-27a targets, such as *SEMA6A*, negatively correlate with miR-27a expression in OS clinical samples.

## RESULTS

### miR-27a and miR-27a* expression in OS cells

**Figure 1:**
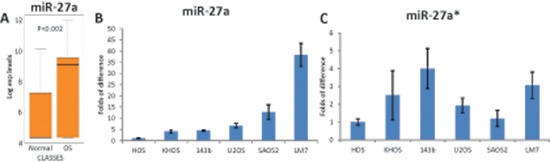
Relative expression of miR-27a in OS, healthy bones and different OS cell lines. Relative expression of miR-27a* in different OS cell lines **(A)** A box plot of miR-27a expression in OS and healthy bones (Normal) according to our published microarray data [19]. Endogenous levels of **(B)** miR-27a and **(C)** miR-27a* in OS cell lines were measured by qRT-PCR and normalized to snoRNA U6. Error bars are SD. Experiments in (B) and (C) were conducted 3 times in triplicates and a representative figures are shown.

We previously reported that higher expression of *miR-27a* is associated with clinical metastatic disease in OS patients. We also reanalyzed our data [19] and found that *miR-27a* is upregulated in OS relative to healthy bones (Figure 1A). To validate that *miR-27a* is pro-metastatic in OS, we first examined the expression of miR-27a and miR-27a*, a microRNA pair derived from a single precursor, in different human OS cell lines using qRT-PCR. We found differential expression of miR-27a and miR-27a* in all OS cells with a prominently high level of expression in LM7 cells (Figure 1B, 1C). Since LM7 cells are of high metastatic potential and were originally derived from low metastatic potential SAOS2 cells [20], we mainly studied miR-27a and miR-27a* pro-metastatic effects in these two cell lines.

### miR-27a and miR-27a* co-overexpression promotes metastatic properties of SAOS2 cells

**Figure 2:**
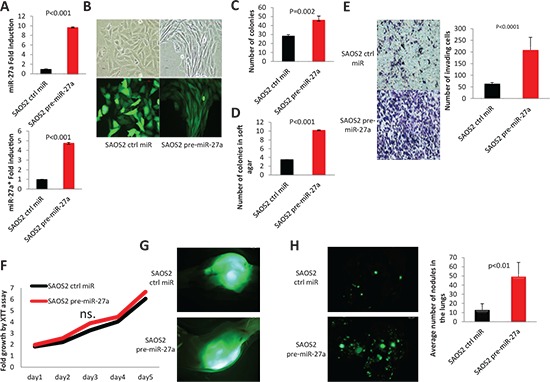
Effects of *MIR-27a* gene overexpression on properties of SAOS2 cells **(A)** miR-27a and miR-27a* overexpression in SAOS2 cells is assessed by qRT-PCR. **(B)** Morphology and GFP fluorescence of SAOS2 cells transduced with Lenti-miR-27a and Lenti-Contr-miR. Images were obtained using 10x magnification. **(C)** Colony formation assay. **(D)** Soft agar colony formation assay. **(E)** Matrigel invasion assay. Images were obtained using 10x magnification. **(F)** XTT proliferation assay. OD_500_ was measured for each sample each 24 hours and normalized to the first measurement for each sample. n.s. – no statistically significant difference at any time point as estimated by the Student's *t*-test. **(G)** and **(H)** NOD/SCID mice were sacrificed 10 weeks after injecting SAOS2-Contr-miR or SAOS2-miR-27a cells into the tibia (IT). Six mice were assigned to each group. (G) Representative images of GFP-labeled injected SAOS2-Contr-miR and SAOS2-miR-27a cells in mouse tibia 10 weeks after IT injections; Stereoscope objective magnification – 0.7x. (H) Representative images and quantification of lung metastases in the same mice; objective magnification – 0.7x. *P* value according to the Student's *t*-test is indicated wherever is relevant. Error bars are SD. In (A), (C), (D), (E) and (F) each experiment was conducted at least 3 times in triplicate and a representative picture is shown.

To study the pro-metastatic roles of miR-27a and miR-27a*, we overexpressed *MIR-27a* gene, which encodes for both miR-27a and miR-27a*, using a lentiviral vector that expresses a *GFP* reporter in SAOS2 cells and studied its metastatic traits both *in vitro* and *in vivo* (Figure 2A). A lentivirus, which carries a control *miR* gene was used as a control. Notably, SAOS2 cells underwent morphological changes upon *MIR-27a* gene overexpression and acquired more elongated shape resembling LM7 cells morphology (Figure 2B). miR-27a and miR-27a* co-overexpression also led to a significant increase in the number of colonies in colony formation and soft agar colony formation assays (Figure 2C, 2D) suggesting pro-survival properties of these miRNAs. Overexpression of these miRs also significantly increased SAOS2 cell invasiveness *in vitro* as assessed by Matrigel invasion assay (Figure 2E) but had no effect on SAOS2 cell proliferation in an XTT proliferation assay (Figure 2F).

Next, we set to determine the effect of *MIR-27a* gene expression on metastatic traits of SAOS2 cells *in vivo*. Remarkably, *MIR-27a*-expressing SAOS2 cells formed significantly more lungs metastases in NOD/SCID mice upon orthotropic intratibial (IT) injections than control SAOS2 cells (Figure 2G, 2H). Thus, miR-27a and miR-27a* co-overexpression boosts SAOS2 cell metastatic properties suggesting pro-metastatic functions of these miRNAs.

### miR-27a and miR-27a* inactivation by sponges reduces metastatic properties of LM7 cells

**Figure 3:**
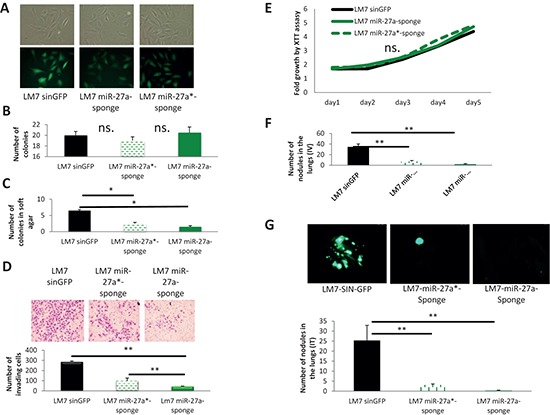
Effects of miR-27a and miR-27a* inactivation on properties of LM7 cells **(A)** GFP fluorescence and morphology of LM7-SIN-GFP, LM7-miR-27a-Sponge and LM7-miR-27a*-Sponge cells; images were obtained using 10x magnification. **(B)** Colony formation assay. **(C)** Soft agar colony formation assay. **(D)** Matrigel invasion assay. Images were obtained using 10x magnification. **(E)** XTT proliferation assay. OD_500_ was measured for each sample each 24 hours and normalized to the first measurement for each sample. n.s. – no statistically significant difference at any time point as estimated by the Student's *t*-test. **(F)** Lung metastases in NOD/SCID mice after IV injections – quantification. Mice (*n* = 6/group) were sacrificed 4 weeks after IV injections. **(G)** Lung metastases in IT injected mice formed by injected GFP-labeled LM7-SIN-GFP, LM7-miR-27a-Sponge and LM7-miR-27a*-Sponge cells – representative pictures were obtained using stereoscope using objective magnification of 0.7x. Mice (*n* = 6/group) were sacrificed 10 weeks after IT injections. Error bars are SD. In (B), (C), (D) and (E) each experiment was conducted at least 3 times in triplicate and a representative picture is shown. The statistical significance was estimated by the Student's *t*-test for all experiments related to the control (LM7 sinGFP); * indicates *P* value < 0.05, ** – *P* value < 0.01.

Our results obtained from overexpressing *MIR-27a* gene prompted us to test whether miR-27a and miR-27a* inhibition will affect the metastatic traits of OS cells. MiRNA sponges were developed as an efficient tool for miRNAs inactivation [21]. Here, we applied the miRNA sponge technology to further prove the roles of miR-27a and miR-27a* in metastatic properties of LM7 cells. To this end, LM7 cells were transduced with a control lentivirus, which carries GFP with a control sponge at 3′-UTR, or with a lentivirus, carrying GFP with either miR-27a or miR-27a* sponge at the 3′-UTR. Transduced LM7 cells were designated LM7-SIN-GFP, LM7-miR-27a-Sponge and LM7-miR-27a*-Sponge correspondingly. All sponges were efficiently overexpressed as confirmed by GFP expression (Figure 3A). Although LM7-miR-27a-Sponge and LM7-miR-27a*-Sponge cells were not significantly different from LM7-SIN-GFP cells in the standard colony formation assay (Figure 3B), they formed significantly fewer colonies than control LM7-SIN-GFP cells in the more rigorous soft agar colony formation assay (Figure 3C). These data suggest that inactivation of miR-27a as well as miR-27a* reduces the oncogenicity of LM7 cells.

In a Matrigel invasion assay, LM7-miR-27a-Sponge and LM7-miR-27a*-Sponge cells displayed significantly lower invasive capabilities than LM7-SIN-GFP cells *in vitro* (Figure 3D). No effect of miR-27a or miR-27a* inactivation on LM7 cells proliferation was found in the XTT proliferation assay (Figure 3E).

In order to directly assess the effect of miR-27a and miR-27a* inactivation on metastatic properties of LM7 cells *in vivo*, we injected these cells in the tail vein (IV) or orthotropically in the tibia (IT) of NOD/SCID mice. LM7-miR-27a-sponge as well as LM7-miR-27a*-Sponge cells formed significantly fewer metastatic foci in the lungs of mice than LM7-SIN-GFP cells in both types of injections (Figure 3F, 3G). Of note, no differences were observed in the size of primary tumors in the case of IT injections (data not shown). Hence, inactivation of miR-27a or miR-27a* significantly reduce the metastatic ability of LM7 cells without affecting primary tumor growth. Therefore, our findings suggest that both miRNAs – miR-27a and miR-27a* – possess pro-metastatic functions in OS cells.

### miR-27a* inactivation leads to the suppression of metastatic potential in HOS and KHOS osteosarcoma cells

**Figure 4:**
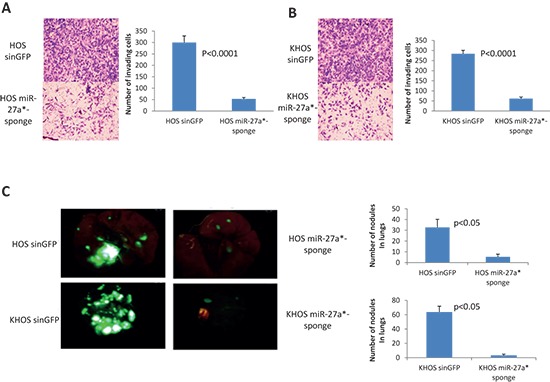
Effects of miR-27a* inactivation in HOS and KHOS cells **(A, B)** *In vitro* invasion assay of HOS-SIN-GFP and HOS-miR-27a*-Sponge cells (A) and of KHOS-SIN-GFP and KHOS-miR-27a*-Sponge cells (B) Images were obtained using 10x magnification. All experiments, which are presented on figures (A) and (B), are conducted at least 3 times in triplicates. **(C)** Lung metastases in NOD/SCID mice (*n* = 5/group) 4 weeks after IV injections with HOS-SIN-GFP, HOS-miR-27a*-Sponge, KHOS-SIN-GFP and KHOS-miR-27a*-Sponge cells – representative pictures were obtained using stereoscope using objective magnification of 0.7x. *P* value according to the Student's *t*-test is indicated wherever is relevant. Error bars are SD. In (A) and (B) each experiment was conducted at least 3 times in triplicate and a representative picture is shown.

We previously showed that overexpression of MIR-27a gene in HOS cells render them more metastatic [19]. In order to further confirm pro-metastatic functions of miR-27a* in OS cells, and in order to show that these effects are not cell line specific, we examined the effects of miR-27a* inactivation by miR-27a*-Sponge in HOS and KHOS cells. We transduced HOS and KHOS cells with the control lentivirus and designated these cells HOS-SIN-GFP and KHOS-SIN-GFP, respectively. We also transduced HOS and KHOS cells with the lentivirus, which carries miR-27a*-Sponge, and designated these cells HOS-miR-27a*-Sponge and KHOS-miR-27a*-Sponge, respectively. HOS-miR-27a*-Sponge and KHOS-miR-27a*-Sponge cells displayed dramatically reduced invasiveness in comparison to corresponding control cells as assessed by the *in vitro* invasion assay (Figure 4A, 4B). Furthermore, HOS-miR-27a*-Sponge and KHOS-miR-27a*-Sponge cells form a significantly reduced number of lung metastases as compared with HOS-SIN-GFP and KHOS-SIN-GFP, respectively (Figure 4C). These data further indicate that miR-27a*, similar to miR-27a, has pro-metastatic functions in broad spectrum of OS cell lines.

### *CBFA2T3* is a target of miR-27a and miR-27a*

**Figure 5:**
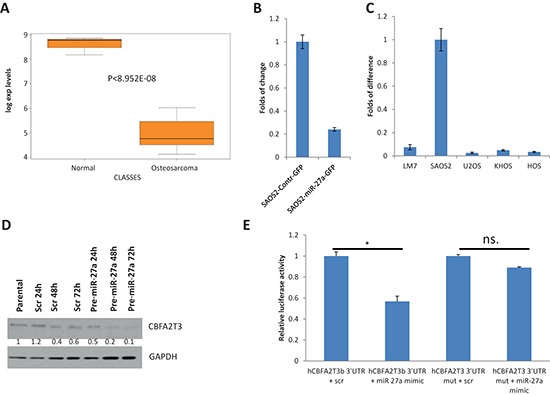
*CBFA2T3* is a target of miR-27a and miR-27a* **(A)** A box plot of *CBFA2T3* expression in OS and healthy bones (Normal) according to our published Affymetrix microarray data [19]. **(B)** Effect of *miR-27a* gene overexpression in SAOS2 cells on endogenous *CBFA2T3* expression as assessed by qRT-PCR. **(C)** Relative expression of CBFA2T3 in different OS cell lines as assessed by qRT-PCR. **(D)** Effect of transfection of HEK293T cells with synthetic scrambled pre-miR or with synthetic pre-miR-27a on endogenous *CBFA2T3* expression at the protein level as assessed by western blot with anti-CBFA2T3 antibody. Quantification of the CBFA2T3 expression shown below was performed by Image J program. Intensity of CBFA2T3 bands was normalized to intensity of GAPDH bands. CBFA2T3 level in the first not transfected sample was taken as 1. **(E)** Luciferase assay of the effect of miR-27a on expression of *firefly luciferase* gene, which contains a *CBFA2T3* 3′-UTR fragment with wild type (CBFA2T3 3′-UTR) or mutant (CBFA2T3 3′-UTR mut) miR-27a binding site. Transfections of HEK293T cells were conducted in triplicates as described in the “Materials and Methods” section. 48 hours after transfections cells were lysed and luciferase activities were measured. Relative firefly luciferase activity normalized to renilla luciferase activity is shown on the figure. Error bars are SD. In (B), (C) and (E) each experiment was conducted at least 3 times in triplicate and a representative picture is shown. * indicates *P* value < 0.05 and n.s. – no statistical significance as estimated by the Student's *t*-test.

In order to address the molecular mechanism underlying the pro-metastatic functions of miR-27a and miR-27a*, we studied the effect of overexpressing and inactivation of these miRNAs on expression of several putative miR-27a targets, which were identified as downregulated in OS versus healthy bones [19]. A candidate gene, which is downregulated in OS (Figure 5A) and is putatively targeted by miR-27a as predicted by TargetScan, is *CBFA2T3*. Indeed, gene expression of *CBFA2T3* was altered by *MIR-27a* gene overexpression (Figure 5B). Therefore, we decided to study this miR-27a target in greater details.

We first examined *CBFA2T3* expression in several OS cell lines and found that its expression is very low in most OS cell lines but high in SAOS2 cells (Figure 5B). Notably, *CBFA2T3* expression was inversely associated with miR-27a and miR-27a* expression in SAOS2 and LM7 cells (Figure 1B and 5C). This is consistent with the possibility that regulation of *CBFA2T3* expression by miR-27a and miR-27a* may mediate effects of these miRNAs on metastatic properties of SAOS2 and LM7 cells.

To further examine that miR-27a directly targets *CBFA2T3*, we tested the effect of miR-27a on CBFA2T3 protein expression. To this end, we examined the effect of transfecting synthetic *miR-27a* precursor on endogenous CBFA2T3 expression in HEK293T cells. As shown in Figure 5D, CBFA2T3 protein levels were significantly downregulated 48 and 72 hours after transfection in comparison to HEK293T cells transfected with scrambled miRNA precursor.

In order to further clarify whether *CBFA2T3* is a direct target of miR-27a we cloned a fragment of 3′-UTR of *CBFA2T3*, which harbors a predicted miR-27a binding site, in the 3′-UTR of *firefly luciferase* gene and designated this construct CBFA2T3–3′-UTR. Synthetic miR-27a precursor (containing miR-27a and miR-27a*) significantly reduces firefly luciferase activity when co-transfected with CBFA2T3–3′-UTR while a mutation, which affects miR-27a binding site, nullifies this effect (Figure 5E). Additionally, we observed that miR-27a* alone can reduce luciferase activity of CBFA2T3–3′-UTR (data not shown). Hence, miR-27a and miR-27a* can directly regulate *CBFA2T3* expression through their binding sites in the 3′-UTR of *CBFA2T3*.

### *CBFA2T3* is downregulated in majority of OS samples

Based on our data suggesting that *CBFA2T3* is a potential target of miR-27a and miR-27a* and the fact that *CBFA2T3* is genetically altered and/or has tumor suppressive properties in different cancer types [22–31], we decided to address a possible tumor suppressive role of *CBFA2T3* in osteosarcomagenesis.

**Figure 6:**
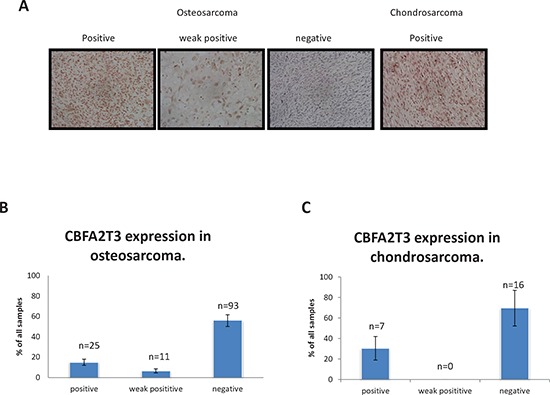
Immunohistochemical assessment of CBFA2T3 expression in OS and chondrosarcomas **(A)** Representative images of immunohistochemical staining of CBFA2T3 expression showing nuclear staining. Images were obtained using 40x magnification. **(B)** Percentage of OS samples, which are positive, weakly positive or negative for CBFA2T3 expression according to immunohistochemical staining (*n* = 129). **(C)** Percentage of chondrosarcoma samples, which are positive, weakly positive or negative for CBFA2T3 expression according to immunohistochemical staining (*n* = 23). Error bars in (B) and (C) are SEM.

As mentioned above, *CBFA2T3* is downregulated in OS according to our Affymetrix microarray data [19]. To further validate these findings, we applied immunohistochemistry on OS and chondrosarcoma tissue microarrays using anti-CBFA2T3 antibody. The CBFA2T3 protein is mainly expressed in the nucleus and therefore we scored its expression based on strong nuclear staining (positive), moderate (weak positive) or absent (negative), as presented in Figure 6A. We found that the CBFA2T3 protein is undetectable or reduced in majority of OS samples (80.6% ± 7.9%; Figure 6B) and chondrosarcoma samples (69.6% ± 17.4%; Figure 6C). This finding is consistent with the possibility that *CBFA2T3* acts as a tumor suppressor in OS.

### *CBFA2T3* acts as a tumor suppressor in OS cells and its overexpression counteracts *miR-27a* overexpression in SAOS2 cells

**Figure 7:**
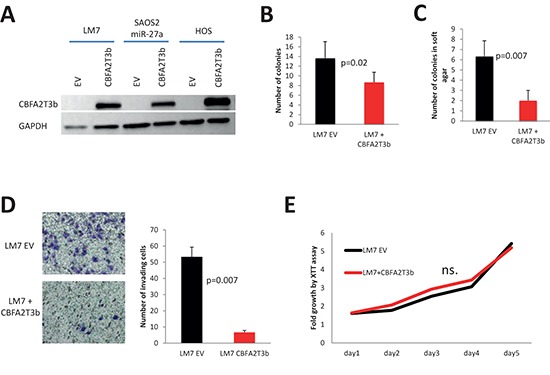
Effect of *CBFA2T3* overexpression in LM7 cells **(A)** overexpression of myc-tagged CBFA2T3 in LM7, SAOS2-miR-27a and HOS cells as assessed by western blot with the anti-myc antibody. **(B)** Colony formation assay. **(C)** Soft agar colony formation assay. **(D)** *in vitro* cells invasion assay (objective magnification – 10x); **(E)** XTT proliferation assay. OD_500_ was measured for each sample each 24 hours and normalized to the first measurement for each sample. n.s. – no statistically significant difference at any time point as estimated by the Student's *t*-test. Error bars are SD. In (B), (C), (D) and (E) each experiment was conducted at least 3 times in triplicate and a representative picture is shown. *P* value according to the Student's *t*-test is indicated wherever is relevant.

**Figure 8:**
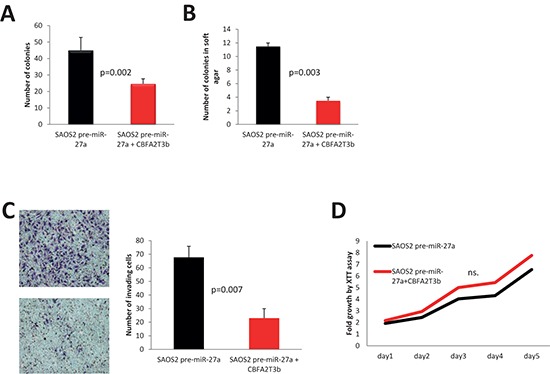
Effects of *CBFA2T3* overexpression in SAOS2-miR-27a cells **(A)** colony formation assay; **(B)** soft agar colony formation assay. **(C)** *in vitro* cells invasion assay. Images were obtained using 100x magnification. **(D)** XTT proliferation assay. OD_500_ was measured for each sample each 24 hours and normalized to the first measurement for each sample. n.s. – no statistically significant difference at any time point as estimated by the Student's *t*-test. All experiments were conducted at least 3 times in triplicates. Error bars are SD. *P* value according to the Student's *t*-test is indicated wherever is relevant.

**Figure 9:**
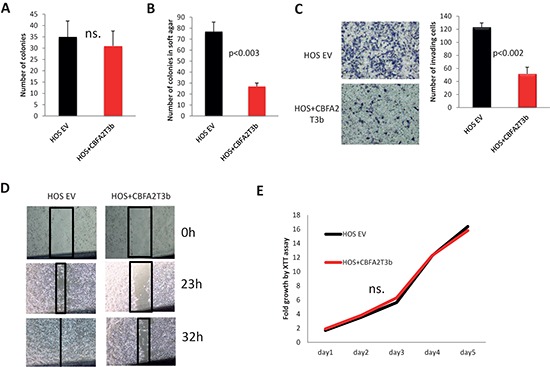
Effects of *CBFA2T3* overexpression in HOS cells **(A)** Colony formation assay. **(B)** Soft agar colony formation assay. **(C)** *in vitro* cells invasion assay; Images were obtained using 100x magnification. **(D)** Wound healing assay. Images were obtained using 4x magnification. **(E)** XTT proliferation assay. OD_500_ was measured for each sample each 24 hours and normalized to the first measurement for each sample. n.s. – no statistically significant difference at any time point as estimated by the Student's *t*-test. All experiments were conducted at least 3 times in triplicates. Error bars are SD. *P* value according to the Student's *t*-test is indicated wherever is relevant.

In order to directly address the question whether CBFA2T3 functions as a tumor suppressor in OS, HOS, LM7 and SAOS2 cells were transduced with a lentivirus, which carries myc-tagged *CBFA2T3* (Figure 7A). In almost all cell lines with the exception of HOS cells, we observed significant decrease in the number of colonies upon *CBFA2T3* overexpression in colony formation assays (Figure 7B, 8A, 9A). *CBFA2T3* overexpression led, also, to a significant decrease in the number of colonies in soft agar colony formation assay (Figure 7C, 8B, 9B). In addition, *CBFA2T3* overexpression attenuated OS cells invasiveness and motility (Figure 7D, 8C, 9C, 9D). No effect on OS cells proliferation was observed as assessed by the XTT assay (Figure 7E, 8D, 9E). Interestingly, restoration of *CBFA2T3* expression in SAOS2 cells, which overexpress miR-27a and miR-27a*, counteracts miR-27a and miR-27a* effects and reduces SAOS2 cells survival and invasiveness but not its proliferation (Figure 8A, 8B, 8C, 8D). These data suggest that miR-27a and miR-27a* regulation of *CBFA2T3* expression may mediate miR-27a and miR-27a* pro-metastatic actions. Thus, *CBFA2T3* overexpression limits survival, invasion and motility but not proliferation of OS cells *in vitro*, consistent with observed phenotypes of miR-27a (Figure 2, 3).

**Figure 10:**
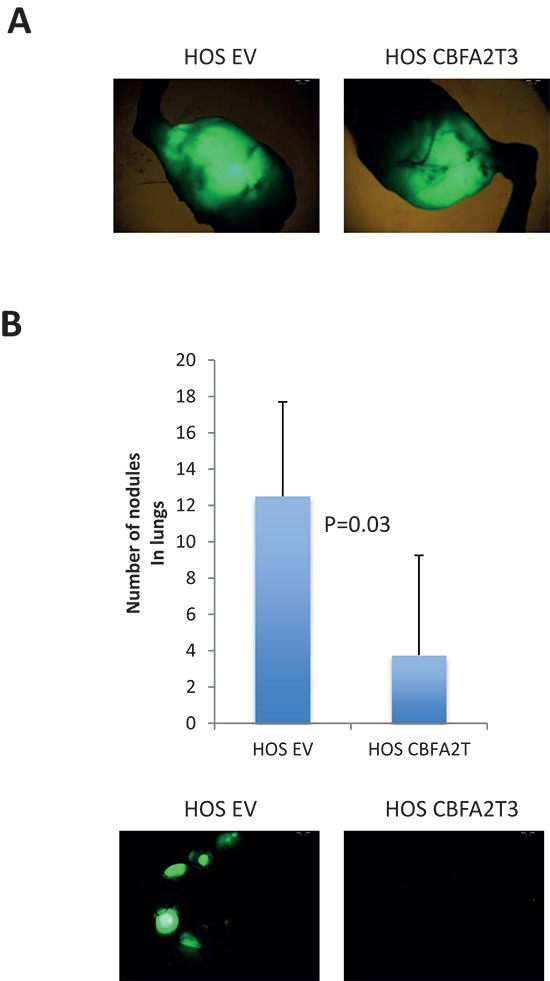
Effect of *CBFA2T3* overexpression in HOS cells on primary tumors and lung metastases NOD/SCID mice (*n* = 5/group) were sacrificed 10 weeks after IT injecting GFP-labeled HOS-EV and HOS-CBFA2T3 cells. **(A)** Representative images of primary tumors of mice 10 weeks after IT injections were obtained using stereoscope using objective magnification of 0.7x. **(B)** Lung metastases in the same mice – quantification and representative pictures (objective magnification – 0.7x). Error bars are SD. The *P* value is indicated according to the Student's *t*-test.

We next addressed whether overexpression of *CBFA2T3* inhibits metastatic traits *in vivo*. Control or CBFA2T3-expressing HOS cells were IT-injected and lung metastatic foci were quantified. We found that indeed *CBFA2T3* overexpression decreased metastatic potential of HOS cells *in vivo* without affecting growth of primary tumors (Figure 10A, 10B). Therefore, *CBFA2T3* exerts anti-metastatic properties in OS cells.

Overall, our data indicate that *CBFA2T3* has a tumor suppressive, anti-metastatic role in OS cells and regulation of *CBFA2T3* expression by miR-27a and miR-27a* might be accountable for the pro-metastatic action of these miRNAs.

### miR-27a and miR-27a* regulate expression of other OS-related genes

To gain more insights into the pro-metastatic function of *miR-27a*, we reanalyzed our published OS mRNA profiling [19] addressing the question whether *CBFA2T3* expression negatively correlates with *miR-27a* expression in OS clinical samples but found no significant correlation (*R* = −0.28) perhaps since *miR-27a* might target *CBFA2T3* translation rather than its mRNA degradation. It is also possible that in addition to *miR-27a* other factors might affect *CBFA2T3* expression in OS. It was, therefore, interesting to investigate whether there were other *miR-27a* targets which expression has significant negative correlation with *miR-27a* expression in OS samples. To this end, we reanalyzed our published OS mRNA profiling [19] to generate a list of all genes, which expression correlates with *miR-27a* expression in OS clinical samples (Supplementary Table 3). Interestingly, among these genes there was *SEMA6A* (*R* = −0.772, *p* < 0.01), which is an established *miR-27a/b* target [32]. Notably, *SEMA6A* has a higher level of expression in SAOS2 cells than in LM7 cells (Supplementary Figure 1). In other words, *SEMA6A* expression is inversely associated with the metastatic potential and miR-27a in SAOS2 and LM7 cells suggesting that regulation of *SEMA6A* expression might contribute to the difference in the metastatic potential and pro-metastatic effects of miR-27a in these cells.

## DISCUSSION

We recently reported that high levels of *MIR-27a* expression are associated with metastatic OS and *MIR-27a* gene overexpression boosts metastatic properties of HOS cells [19]. This gene encodes two miRNAs – majorly presented miR-27a and low-presented miR-27a*. The oncogenic role of one of these miRNAs – miR-27a in OS cells was confirmed later by an independent research group [33]. Here we provided additional data supporting pro-metastatic function of *MIR-27a* gene in OS and, for the first time, demonstrated that both abundant miR-27a and low expressed miR-27a* enhance the metastatic potential of OS cells.

Recently, Wu X. and colleagues published data suggesting that miR-27a* functions as a tumor suppressor in head and neck squamous cell carcinoma by targeting the EGFR signaling axis [16]. They also reported tumor suppressive effects of miR-27a* overexpression in cell lines derived from other solid tumors – PC3 (prostate), HEC1A (endometrial), MIA PaCa (pancreas), and MDA-MB468 (breast) [16]. Similarly, other research group found that miR-27a exerts tumor suppressive properties by targeting EGRF in H1299 cells (non-small cell lung carcinoma) [34]. By contrast, Lerner M and colleagues demonstrated that miR-27a promotes cell cycle and genome instability by targeting *FBW7/hCDC4* tumor suppressor gene [35], which suggests oncogenic properties of miR-27a. These discrepancies among data of different research group may be explained through either fundamental differences between OS and other types of cancer or technicalities of conducted experiments. In fact, miRNAs, which exert oncogenic or tumor suppressive properties depending on experimental settings, are known. For example, B-cell specific *miR-29a/b* overexpression leads to development of CD5-positive B-cell malignancy in mice [36]. On the other hand, *miR-29s* act as tumor suppressors in other types of cancer [37–39]. Thus, the same miRNA may have oncogenic or tumor suppressor properties depending on cellular context which can be explained by expression of different targets of the miRNA in the different cell types.

Interestingly, miR-27a and miR-27a* expression levels indicate different prognosis for survival depending on the type of cancer. We used on-line available datasets and tool for this analysis [40]. Although no data available for OS, high levels of miR-27a expression predict poor survival for patients with nasopharyngeal carcinoma (FDR < 0.036) and early breast cancer (FDR < 0.036). Strikingly, high levels of miR-27a expression give favorable prognosis for survival of patients with high risk ER+ breast cancer (FDR < 0.036). miR-27a expression does not predict survival for patients with hepatocellular carcinoma, prostate cancer, ovarian carcinoma and esophageal adenocarcinoma and squamous cell carcinoma. Intriguingly, high levels of miR-27a* expression also give strong favorable prognosis for survival of patients with high risk ER+ breast cancer (FDR < 0.00042) while do not predict survival of patients with nasopharyngeal carcinoma. These data also support the idea that miR-27a and miR-27a* mode of action strongly depends on the type and even subtype of cancer.

Our findings, which suggest that *CBFA2T3* expression is regulated by both miR-27a and miR-27a* as well as tumor suppressive properties of *CBFA2T3* in OS cells, provide an insight in possible mechanisms underlying the pro-metastatic function of *miR-27a* in OS cells. *CBFA2T3* is affected by different chromosomal aberrations in multiple hematopoietic malignancies [23–31]. CBFA2T3 functions as a transcriptional co-repressor at the molecular level [22, 41–44]. In addition, CBFA2T3 can also bind RNA [44] and function as a dual protein kinase A anchoring protein [46, 47]. Loss of heterozygosity in the genomic region encompassing *CBFA2T3* gene occurs in half of all breast cancer cases and *in vitro* data suggest its tumor suppression function [22, 31]. Of note, obligatory *CBFA2T3* deficient mice do not develop malignancies but have complex defects in the hematopoietic stem cells compartment [48]. The latter is still consistent with a possible role of *CBFA2T3* dysfunction in hematopoietic malignancies. Although *CBFA2T3* deficient mice do not develop malignancies themselves it is possible that *CBFA2T3* deficiency facilitates development and changes aggressiveness of different types of cancer. Our results provide evidence, for the first time, that *CBFA2T3* acts as a tumor suppressor in OS development and progression and can be post-transcriptionally regulated by the pro-metastatic *MIR-27a* gene.

Our analysis revealed no correlation between *CBFA2T3* and *MIR-27a* expression in OS clinical samples. The lack of correlation could stem from the possibility that miR-27a represses *CBFA2T3* translation rather than facilitates mRNA degradation. It is also possible that *CBFA2T3* expression in OS is regulated by other factors, for example, genetic alterations as in hematopoietic malignancies. This finding prompted us to search for miR-27a targets, which expression is inversely correlated with *MIR-27a* expression in OS clinical samples. Our search indicated *SEMA6A* (*Semaphorin 6A*) as a potential miR-27a target; its expression is inversely correlated with *MIR-27a* expression in OS clinical samples and it was previously reported to be a direct target of miR-27a/b [32]. *SEMA6A* can regulate cells migration through physical interaction with its receptors - plexin-A2 and plexin-A4 [49–51]. This makes possible its participation in the metastatic process, although this possibility needs to be tested. Interestingly, CBFA2T3 binds plexin-A1 and plexin-A3 but not plexin-B1 while CBFA2T3 binding to plexin-A2 and plexin-A4 was not determined [47]. It is thus possible that miR-27a targets both CBFA2T3 and SEMA6A modulating the same signaling pathway.

Overall, our findings of miR-27a and miR-27a* acting as pro-metastatic miRNAs contribute to a growing body of evidence that star miRNAs, which were originally mostly viewed as degraded byproducts [52], are functional and might have driver function in the tumorigenesis process. We also for the first time provide evidence that *CBFA2T3* acts as a tumor suppressor in OS cells suggesting that deregulated *CBFA2T3* expression by miR-27a and miR-27a*, at least in part, is accountable for their pro-metastatic action in OS. The presented data further suggest the potential of miR-27a and miR-27a* pair as diagnostic markers and therapeutic targets for OS.

## MATERIALS AND METHODS

### Cell lines and cell culture

Cell lines (HOS, KHOS, SAOS2, 143B, U2OS and HEK293T) were obtained from the American Type Culture Collection (ATCC, Manassas, VA). LM7 cell line was a gift of Dr. Kleinerman (The University of Texas MD Anderson Cancer Center, USA). The LM7 cell line was derived from SAOS2 cells as a cell line with higher metastatic potential [20]. LM7 and SAOS2 cell lines were grown in RPMI containing 15% heat-inactivated FBS, 2mM L-glutamine, 100 U/ml penicillin and 100 mcg/ml streptomycin. 143B and HEK293T cells were cultured in DMEM with 10% heat-inactivated FBS, 2mM L-glutamine, 100 U/ml penicillin and 100 mcg/ml streptomycin. The other cell lines were grown in RPMI containing 10% heat-inactivated FBS, 2mM L-glutamine, 100U/ml penicillin and 100 mcg/ml streptomycin.

### Plasmids

Lenti-miR-27a was described elsewhere [19]. Lenti-miR-27a carries *miR-27a* gene under the control of CMV promoter for overexpression of both miR-27a and miR-27a*. This construct has also a *GFP* reporter gene.

Lenti-miR-27a-Sponge and Lenti-miR-27a*-Sponge plasmids were generated as described below. Oligonucleotides, indicated in in the Supplementary Table 1, were phosphorylated at 5′-ends and annealed correspondingly to form two duplexes F1:R1 and F2:R2 for each construct. These duplexes were cloned by the triple ligation in SalI and EcoRI restriction sites in the 3′ untranslated region (3′-UTR) of the GFP gene in SIN18-pRLL-hEFIαp-EGFP-WRPE lentiviral vector [53]. SIN18-pRLL-hEFIαp-EGFP-WRPE is hereafter designated as pSIN-GFP. Each sponge has 6 adjacent binding sites for a corresponding microRNA. Both sponges were confirmed by sequencing. SIN-GFP control sponge as well as Lenti-Contr-miR-GFP were gifts from Dr. Ofer Mandelboim (Hebrew University, Jerusalem, Israel).

A fragment of the human *CBFA2T3* 3′-UTR was amplified from human genomic DNA with primers *CBFA2T3*-3′UTR-D and *CBFA2T3*-3′UTR-R containing XbaI restriction sites (Supplementary Table 1). The amplified fragment was digested with XbaI and cloned in the XbaI restriction site of pGL3-Control vector (Promega, USA) in the 3′-UTR of the *Firefly Luciferase* reporter gene. The obtained construct was designated p3′-UTR-CBFA2T3. p3′-UTRmut-CBFA2T3 with mutated the miR-27a-binding site was generated by use of QuikChange II XL Site-Directed Mutagenesis Kit (Stratagene) and primers CBFA2T3–3′-UTR-mut-D and CBFA2T3–3′-UTR-mut-R.

pLNCX2-CBFA2T3, which encodes CBFA2T3b isoform, was a gift from Dr. David Callen (the University of Adelaide and Hanson Institute, Adelaide, Australia) and described elsewhere [22]. pLV-Neo was a gift from Dr. Ittai Ben Porath (the Hebrew University, Jerusalem, Israel). Myc-CBFA2T3 ORF was amplified from pLNCX2-CBFA2T3 with primers CBFA2T3b-ORF-D and CBFA2T3b-ORF-R (Supplementary Table 1). The amplified myc-CBFA2T3 ORF was cloned in pLV-Neo using the Gateway cloning system (Invitrogen). The obtained construct was designated pLV-Neo-CBFA2T3.

### Immunohistochemistry

Osteosarcoma tissue microarray slides (OS802 & OS804 from US Biomax, Inc) were deparaffinized and rehydrated. Antigen retrieval was performed in 10 mM sodium citrate buffer pH 6.0 using pressurized chamber for 2.5 min. Endogenous peroxidase was blocked with 3% H_2_O_2_ for 10 min. The sections were then incubated with blocking solution (CAS Block, Invitrogen, Grand Island, NY) for 30 min to reduce non-specific binding followed by incubation with the primary polyclonal anti-CBFA2T3 antibody (cat#17190–1-AP, ProteinTech) [dilution of 1:100] in humidity chamber for one hour incubation. Slides were subsequently incubated with horseradish peroxidase-conjugated secondary antibody for 30 min. The enzymatic reaction was detected in a freshly prepared 3,3 diamminobenzidine tetrahydrochloride using DAKO Liquid DAB Substrate-Chromogen (Carpinteria, CA) Solution for several minutes at room temperature. The sections were then counterstained with hematoxylin. Negative controls included slides that were incubated with primary antibody alone without secondary antibody or slides that were incubated with secondary antibody alone without primary antibody. The specific nuclear staining scores were as follows: 0 = negative, 1 = Weak positive, 2 = positive.

### Tumor and metastases formation assays in NOD/SCID mice

All animal work was performed in accordance with the guidelines of the Institutional Animal Care and Use Committee of The Hebrew University of Jerusalem under approved protocol. 1 × 10^6^ cells (SAOS2 cells overexpressing *miR-control* or *miR-27a gene*, LM7 cells expressing sinGFP, miR-27a-sponge or miR-27a*-sponge, HOS and KHOS cells expressing sinGFP or miR-27a*-sponge) were washed in PBS and suspended in serum free medium and injected intravenously (IV) or intratibialy (IT) into four- to six-week-old age-matched male NOD/SCID mice to study lung metastasis. For IV experiments, 6 weeks after injecting the cells, the mice were sacrificed and their lungs were examined for micro and macro-metastasis using a fluorescent stereomicroscope (Olympus). For IT experiments, ten weeks after injecting the cells, the mice were sacrificed and their tibias and lungs were examined.

### Luciferase reporter assay CBFA2T3 targeting by miR-27a

HEK293T cells wells of 12 well plate at 50% confluence were cotransfected with 1 mcg of p3′-UTR-CBFA2T3 or p3′-UTRmut-CBFA2T3, 10 ng of a Renilla luciferase expression construct pRL-TK (Promega) and 40 pmoles synthetic miR-27a precursor or scramble miRNA precursor (Life Technologies) using Lipofetamine 2000 (Life Technologies) according to the manufacture instructions. Cells were harvested 48 hours after transfection and assayed with Dual Luciferase Assay (Promega) according to the manufacturer's instructions. Three independent experiments were performed in triplicate.

### Lentiviral packaging and transduction

HEK293T cells were transfected with 4 mcg of a lentiviral plasmid and packaging plasmids – 0.3 mcg of pVSVG and 2.7 mcg of pGAG-Pol. Transfections were conducted with TransIT-LT1 Transfection Reagent (Mirus Bio LLC, USA) according to the manufacture instructions. Medium was changed 16 hours after transfection and lentivirus containing medium was collected 48 and 72 hours after transfection and filtrated from cells through a filter with 0.45 mcm pores. The filtrated medium with lentiviruses was used for lentiviral transduction. OS cells at 50% confluence in wells of 12 well plates were transduced with 1 ml of a corresponding lentivirus in the presence of 6.6 mcg/ml polybrene overnight. Transduction with lentiviruses, which carry GFP gene as a reporter, was conducted 4–6 times and followed by cell sorting to ensure that all transduced OS cells have bright GFP fluorescence. In the case of lentiviruses, which carry a gene for neomycin resistance, selection with 1.5 mg/ml of G418 antibiotic was started 24 hours after transduction and selected G418-resistant OS cells were used in consequent experiments.

### RNA extraction and quantitative reverse transcription – PCR (qRT-PCR)

Total RNA was extracted using the TRIzol Reagent (Cat # 15596–018, Life Technologies) following the manufacture's instruction. The purified total RNA was used for qRT-PCR of miRNAs as described elsewhere [54]. Primers for qRT-PCR of miRNAs are indicated in the Supplementary Table 2. MiRNAs expression was normalized to snoRNA U6 expression. The remaining total RNA was further cleaned with the RNeasy Mini Kit (Qiagen) according to the manufacture protocol including on-column DNase digestion with RNase-Free DNase Set (Qiagen). The cleaned RNA was further used to generate cDNA according to standard procedures for Sybr Green based qRT-PCR of protein encoding genes with primers, which are listed in the Supplementary Table 2. Protein-coding genes expression was normalized to *UBC* or *GAPDH* expression.

### Matrigel invasion assay

Blind well chemotaxis chambers with 13-mm diameter filters were used. Polyvinylpyrroliodone-free polycarbonate filters, 8 μm pore size (Costar Scientific Co, Cambridge, MA), were coated with basement membrane Matrigel (25 μg per filter). Cells (2 × 10^5^) suspended in DMEM containing 0.1% bovine serum albumin, were added to the upper chamber. Conditioned medium of NIH3T3 fibroblasts was placed in the lower chamber. Assays were carried out at 37 degrees in 5% CO2. Over 90% of the cells were attached to the filter after seven hours of incubation. After incubation, the upper surface of the filter was freed of the cells by using a cotton swab. Cells that passed through the filter to the bottom side were fixed in methanol and then stained by Geimsa. Each triplicate assay was performed three times. Invading cells were counted in ten representative light-microscopy fields with 10x magnification.

### Scratch wound healing assay

Cells were incubated with serum free medium. Images were acquired directly after scratching (0 h) and after 8 h, 24 h and 32 h. Representative images were obtained using inverted light microscope with 4x objective lens.

### Cell proliferation assay

Cells (1.5–2.5 × 10^3^) were plated in 96-well plate for 24 hour intervals, and were analyzed using an XTT (sodium 3′-[1-(phenylaminocarbonyl)-3,4-tetrazolium]-bis (4-methoxy-6-nitro) benzene sulfonic acid hydrate) proliferation assay according to the manufacturers instruction (Biological industries, Israel).

### Colony formation assay

Cells were plated at a density of 300 cells/well in a 6 wells plate in triplicate. After 1–2 weeks the cells were fixed with 70% Ethanol, stained with Giemsa and counted. The test was performed in triplicates and performed twice.

### Soft agar colony formation assay

Cells were plated between two layers of agarose. The lower agar was prepared by melting 1% nobel Agar in water at 42°C and mixed with an equal volume of RPMI 2x containing 20% FCS. The plates were kept at 4°C. The top layer was prepared by melting nobel agar 0.7% in water that was mixed with an equal volume of RPMI 2x containing 20% FCS. Cells were added to this mix and distributed as 2000 cells/well in triplicate. This solution was then gently poured on the base layer. The plates were incubated at 37°C for 14–28 days. Colonies were detected by Crystal Violet staining and then counted.

### Western blot analysis

Cells were lysed using lysis buffer containing 50 mM Tris (pH 7.5), 150 mM NaCl, 10% glycerol, 0.5% Nonidet P-40, and protease inhibitors (1:100). Lysates were resolved on SDS/PAGE. Immunoblot analyses were performed using the following antibodies: anti-myc (Cat#sc-28199, Santa Cruz Biotechnology), anti-GAPDH (Cat#2118, Cell Signaling) and anti-CBFA2T3 (cat#17190–1-AP, ProteinTech).

### Statistics

Results of *in vitro* and *in vivo* experiments were expressed as mean ± SD or mean ± SEM. Student's two tailed *t*-test was applied to compare values of test and control samples. E-MTAB-1136 from Array Express repository was used to study the levels of expression of *hsa-miR-27a*, hsa-miR-27a* and *CBFA2T3* in osteosarcoma samples patients and normal counterparts [19]. The two-tailed paired *t*-test (*p* < 0.05) was employed to assess the relative expression levels (BOX LPLOT IMAGE). Pearson Correlation (*p* < 0.05) was applied to find genes, which expression negatively correlates with *hsa-mir-27a* expression. Raw data for microarray analyses used were deposited in ArrayExpress (E-MEXP-3628 and E-MTAB-1136).

## SUPPLEMENTARY FIGURE AND TABLES




