# Supplementary material for: miR-27a and miR-27a* contribute to metastatic properties of osteosarcoma cells
Source: Oncotarget. 2015 Feb 28;6(7):4920–35. doi: 10.18632/oncotarget.3025 (PMC4467124; doi:10.18632/oncotarget.3025)
Supplement: Supplementary file 1 [file oncotarget-06-4920-s001.pdf]

SUPPLEMENTARY FIGURE AND TABLES

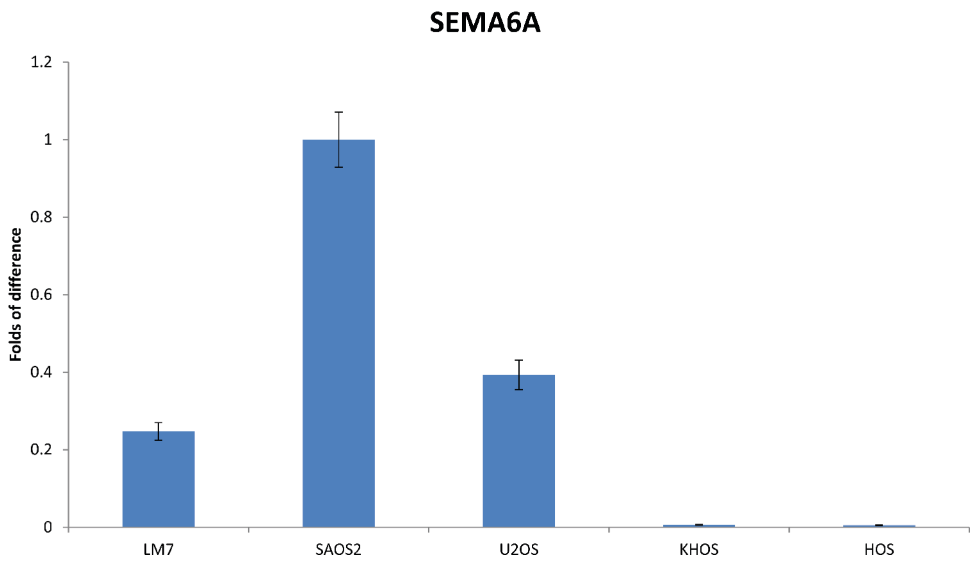

Supplemental Figure 1: Expression of SEMA6A in different OS cell lines. Expression of SEMA6A was assessed by qRT-PCR and normalized to GAPDH expression. Error bars represent SD.

Supplementary Table 1: Oligonucleotides, which were used for cloning

Sponge27a-F1	5′-TCG ACG CGG AAC TTA CCC ACT GTG AAA TGG GCG GAA CTT ACC CAC TGT GAA ATG GGC GGA ACT TAC CCA CTG TGA AA-3′
Sponge27a-R1	5′-AGC TTT TCA CAG TGG GTA AGT TCC GCC CAT TTC ACA GTG GGT AAG TTC CGC CCA TTC ACA GTG GGT AAG TTC CGC G-3′
Sponge27a-F2	5′-AGC TTG CGG AAC TTA CCC ACT GTG AAA TGG GCG GAA CTT ACC CAC TGT GAA ATG GGC GGA ACT TAC CCA CTG TGA AG-3′
Sponge27a-R2	5′-AAT TCT TCA CAG TGG GTA AGT TCC GCC CAT TTC ACA GTG GGT AAG TTC CGC CCA TTC ACA GTG GGT AAG TTC CGC A-3′
Sponge27a*-F1	5′-TCG ACT GCT CAC AAG CCC TAA GCC CTA TGG TGC TCA CAA GCC CTA AGC CCT ATG GTG CTC ACA AGC CCT AAG CCC TA-3′
Sponge27a*-R1	5′-AGC TTA GGG CTT AGG GCT TGT GAG CAC CAT AGG GCT TAG GGC TTG TGA GCA CCA TAG GGC TTA GGG CTT GTG AGC AG-3′
Sponge27a*-F2	5′-AGC TTT GCT CAC AAG CCC TAA GCC CTA TGG TGC TCA CAA GCC CTA AGC CCT ATG GTG CTC ACA AGC CCT AAG CCC TG-3′
Sponge27*-R2	5′-AAT TCA GGG CTT AGG GCT TGT GAG CAC CAT AGG GCT TAG GGC TTG TGA GCA CCA TAG GGC TTA GGG CTT GTG AGC AA-3′
CBFA2T3-3′UTR-D	5′-ATTTCTAGAGGGTTTGTGCCCAGTTA-3′
CBFA2T3-3′UTR-R	5′-ACTTCTAGAAGGAGCACCAGGGCTAT-3′
CBFA2T3-3′UTR-mut-D	5′-ATTTCTAGAGGGTTTGTGCCCAGTTAGAAG-3′
CBFA2T3-3′UTR-mut-R	5′-ACTTCTAGACTTCTAACTGGGCACAAACCC-3′
CBFA2T3b-ORF-D	5′-GGGGACAAGTTTGTACAAAAAAGCAGGCTTCACCATGGAGCAGAAGCTGA TCAGC-3′
CBFA2T3b-ORF-R	5′-GGGGACCACTTTGTACAAGAAAGCTGGGTCTCAGCGGGGCACGGTGTCCA GTGG-3′

Supplementary Table 2: Real-time PCR primers

Primer sequence	Gene or primer name
Forward 5′-ATT TGG GTC GCG GTT CTT G-3′	*UBC*
Reverse 5′-TGC CTT GAC ATT CTC GAT GGT-3′	
Forward 5′-ATG GGG AAG GTG AAG GTC GG-3′	*GAPDH*
Reverse 5′-TGA CGG TGC CAT GGA ATT TG-3′	
Forward 5′-GGG CCT GGT GAA CTC GAC ATT GAC-3′	*CBFA2T3*
Reverse 5′-ACG GCC GCA GAG GGAAGT TGG T-3′	
5′-GCG AGC ACA GAA TTA ATA CGA C-3′	Reverse miR
5′-AGG GCT TAG CTG CTT GTG AGC A-3′	Hsa-miR-27a*
5′-TTC ACA GTG GCT AAG TTC CGC-3′	Hsa-miR-27a
5′-CGC AAG GAT GAC ACG CAA ATT C-3′	U6

Supplementary Table 3: List of genes that correlates with miR-27a expression in OS clinical samples
